# The effects of *Artemisia deserti ethanolic* extract on pathology and function of rat kidney

**Published:** 2014

**Authors:** Ali Noori, Leila Amjad, Fereshteh Yazdani

**Affiliations:** 1 *Department of Biology, Falavarjan Branch, Islamic Azad University, Isfahan, I.R. Iran*; 2 *Young Researchers and Elite Club, Falavarjan Branch, Islamic Azad University, Isfahan, I.R. Iran*

**Keywords:** *Artemisia deserti*, *Extract*, *Kidney*, *Rat*

## Abstract

**Objectives**: Medicinal plants played an important role in human health. The kidney is a major organ for elimination the additional materials of body. Some of metabolic waste products are excreted through the kidneys, give us useful information about kidney health. Therefore, the aim of this research was to study the effects of *A. deserti* flowering tips extract on kidney.

**Materials and** **Methods:** Three groups of animal were studied. Wistar rats were divided into three groups. Group 1 was injected with saline, group 2 and 3 were injected with extract, 100 mg/kg and 200 mg/kg, respectively. The animals were anesthetized, blood samples were collected 2 days after the last injection, then urea, uric acid and creatinine levels were assayed. Also, the kidney histology was studied.

**Results:** No significant changes in urea and uric acid were observed. But, creatinine concentration was changed significantly in group 3 compared to other groups. The extract caused histologic changes in the kidney, including, glomerular atrophy, congestion of inflammatory cells and degeneration of the renal tubules.

**Conclusion**: The results showed that *A. deserti *extract was able to damage the kidney tissue. However, the reason for these histopathological changes remains to be clarified.

## Introduction

Herbal medicine plays an important role in human healthcare and because they belong to the natural resources they are the main ingredient of traditional medicine (De Smet, 2002 ▶). World Health Organization (WHO) estimates that 80% of the world populations currently use herbal drugs for healthcare. Generally these drugs are considered to be free of side effects (Bodhisattwa et al., 2011 ▶).

The genus *Artemisia *is the largest member of Asteraceae, which comprises around 500 species.* Artemisia *species are widely distributed in temperate regions in the northern hemisphere but very sparsely in the southern hemisphere, with less than 10 species there (Garcia et al., 2011 ▶).

These species contain acetylenic compounds, flavonoids, coumarins and terpenoids, specifically sesquiterpene lactones and other constituents. *Artemisia* extract has insecticidal, anti-parasitic, anti-fungal, sedative and anti-cough activity and it used for medicinal, ornamental and culinary purposes (Rustaiyan et al., 2000 ▶; Kazemi et al., 2011 ▶). 

*A.* *deserti* Krasch is a traditional medicinal herb of China. It is presently being cultivated in a commercial scale in China and Vietnam and this is probably due to sesquiterpene lactones compounds (Rustaiyan et al., 2000 ▶). In the study of Rustaiyan et al. (2000) ▶, 16 components were recognized in the oil of aerial parts from *A.deserti*, so that, camphor (45.5%), 1,8-cineole (16.7%), piperiton (8.6%), β*-*pinene (5.7%) and isoborneol (3.2%) were the major components in the oil of *A. deserti*. *A. deserti* oil consists of 5 monoterpene hydrocarbons (8.4%), 9 oxygenated monoterpenes (75.7%) and 2 sesquiterpenes (0.9%). Also, leaf and flower oils of *A.* *deserti *were observed to be rich in oxygenated monoterpenes (68.2% and 59.2% respectively) while oxygenated monoterpenes (37.9%) and sesquiterpenes (33.8%) were the major components in its stem (Kazemi et al., 2011 ▶).

Sesquiterpene lactones constitute a large group of biologically active plant chemicals that have been identified in several plant families such as Asteraceae. Artemisinin is a sesquiterpene lactone that exists in *Artemisia* genus (Chaturvadi, 2011 ▶). Ferreira et al. (2010) ▶ reported that artemisinin was metabolized by the liver CYP450 enzyme. But the pharmacological levels of artemisinin in the blood would decrease significantly after 5-7 days of treatment with the extract. This is due to induction of CYP450 enzyme. Adam et al. (2000) ▶, also reported the presence of alkaloids, flavonoids, sterols, tannins, volatile oils and anthraquinones in aerial parts of the *Artemisia* species. These compounds were considered according to findings of Iriadam et al. (2006) ▶ for detoxification of organs. 

Because of side effects of herbs extracts on organisms (Eweka, 2007 ▶; Atawodi et al., 2010), it was necessary to study the effect of *A. deserti* extract on kidney function. The kidney is the primary organ for clearance and excretion of xenobiotics including drugs from the body. Moreover, electrolyte and water balance are regulated via the kidney. Urea and creatinine are waste products of protein metabolism that are excreted through the kidney. The increase of urea and creatinine is a sign of kidney damage. Although, urea concentration increases due to dehydration, drugs and diet (Ene-ojo et al., 2013 ▶). Creatinine is a product of creatine which is excreted by the kidney and the amount of this biochemical compound in the blood is proportional to the glomerular filtration rate. Uric acid is the metabolic end product of purine metabolism in humans, which is excreted by the kidney. It has antioxidant properties but can also be pro-oxidant, depending on its chemical microenvironment (So and Thorens, 2010 ▶). Histological analysis of kidney tissue and determination of some waste metabolic products excreted via the kidneys provide useful information about the health of this organ. Therefore, the aim of this study was to evaluate the effects of ethanolic extract of *A. deserti* flowers on kidney histology and function.

## Materials and Methods

**Collection of plants**

The flowering tips of *A. deserti* were collected from the west province of Isfahan (Golpaygan heights), Iran, in September 2012. The voucher specimen was deposited at the herbarium of the Research-Institute of Isfahan Forests and Rangelands.

**Preparation of extract**

The flowering tips of *A. deserti* were air-dried under the shade and grounded into fine powder using electric blender. Then, 20 g of flower powder was extracted with 150 mL ethanol 80% by soxhlet extractor for 8 hours. The residue was evaporated by using a rotary evaporator. The dried extracts were stored at 4°C until used. The extracts were dissolved in saline to prepare the doses of 100 and 200 mg/kg body weight rats (Ene-ojo et al., 2013 ▶).

**Animals**

Adult male wistar rats (200-250 g) were obtained from Iran Pasteur Institute and divided into three groups of eight animals each (24 rats). They were maintained under controlled temperature, 12 h light/12 h dark conditions for 1 week before starting the experiments for adaption to laboratory conditions. The procedures in this study were carried out in accordance to the institution's scientific procedures for animals and was approved by the Institutional Animal Care protocol. Animals were randomly divided into the saline injection group (control) and treatment groups (2 and 3). 

The treatment groups were injected intraperitoneally with extract (100, 200 mg/kg body weight, daily) respectively, for 6 days. The animals were anesthetized with ketamine (0.07ml/100kg body weight) and the blood samples were collected 2 days after the last injection. The serum biochemical parameters including urea, uric acid and creatinine were assayed using autoanalyzer (902 Hitachi Automatic Analyzer, Roche, India). Then, the animals were killed and kidney tissue was fixed in 10% formalin, dehydrated in ethanol, cleared in xylene, and embedded in paraffin. Sections were prepared and then stained with Hematoxylin and Eosin (H&E) for photomicroscopic (Olympus, Japan) observation.

**Statistical analysis**

All data are presented as Mean±SEM. Statitical analysis was performed by using SPSS 18 for Windows. Data were analyzed by using a One-Way ANOVA test and significance was considered at p<0.05.

## Results

No significant changes in urea and uric acid were observed between three groups. Although, these parameters were different between groups but these differences were not significant (p>0.05). Whereas serum creatinine was decreased significantly in group 3 (200 mg/kg) compared with other groups (p<0.05, Table 1). 

**Table 1:** Serum biochemical parameters in different groups

Groups	Creatinine (mg/dl) Mean ± SD	Urea (mg/dl) Mean ± SD	Uric Acid (mg/dl) Mean ± SD
Control	0.575± 0.0707	17.75± 2.12	0.775 ± 0.265
Extract (100 mg/kg)	0.537± 0.0517	19.37± 3.11	1.037± 0.370
Extract (200 mg/kg)	0.475± 0.0462*	20.12± 2.90	0.887± 0.383

**Figure 1:**
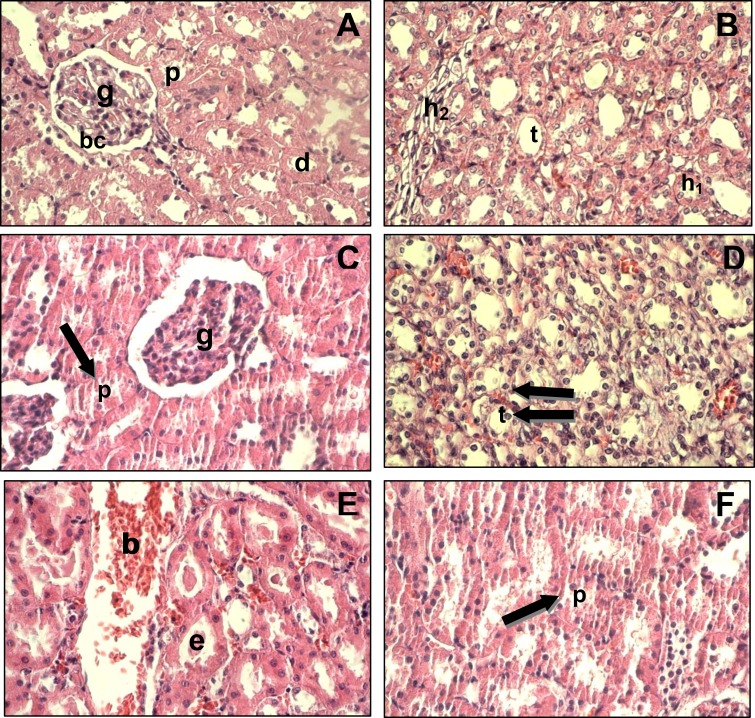
Light microscopy of the rat kidney from control and treatment groups. (A) the kidney cortex of control group shows that the glomerulus, proximal tubules, distal tubules and Bowman ’s capsule were normal; (B) the renal medulla of control group shows the thick and thin segments of ascending and descending loop of Henle, and collecting tubule were normal; (C) the kidney cortex of treatment groups shows the degeneration in the wall of renal tubules (arrow) and the atrophied glomeruli; (D) the renal medulla of treatment groups shows the degeneration in the lining epithelial cells of renal tubules and the nucleus of their cells were released (arrow); (E) the kidney cortex of treatment groups shows several renal tubules contain dense eosinophilic material and eosinophilic fluid or blood; (F) the kidney cortex of treatment groups shows the degeneration in the wall of renal tubules (arrow); (g, glomerulus; bc, Bowman-^'^s capsule; p, proximal tubules; d, distal tubules; h_1_, thick segment of henle; h_2_, thin segment of henle; t, collecting tubule; b, eosinophilic fluid or blood; e, eosinophilic material) (×400).

Histological studies showed that the kidney tissue was normal in the control group. But, the treating animals with *A. deserti* extract showed significant histopathological alterations. These alterations were included the degeneration in the wall of proximal and distal tubules, atrophied glomeruli and swallowed endothelial cells. Also, some tubules were contained dense eosinophilic material and fluid or blood. Moreover, the inflammatory cells were observed in the kidney tissue (Figure 1). The results were showed that these histopathological alterations were increased in group 3 (200 mg/kg) compared with group 2 (100 mg/kg).

## Discussion

Results of this study showed that extract of *A. deserti* produced histopathological alterations in the kidney of rats. Also, serum creatinine concentration was decreased significantly in group 3 (200 mg/kg) that is probably due to the extract effect on liver function (Safa et al., 2005). Whereas, this result was not similar to Iriadam et al. (2006) ▶ findings, as they reported that there were no significant histopathological changes in the kidney after treatment of rabbits by aqueous extract of *Artemisia herba alba* aerial parts at 80 mg/kg. In another study, no significant alterations were observed in the kidney of the rats in a 2% *Artemisia abyssinica *leaves diet. Whereas, the significant alterations were observed in all rats kidney were fed on a 10% diet of extract that was associated with a decrease in the amount of urea. These data indicate that the sensitivity of the animals to plant materials was dependent to the active component and concentration added to the diet (Adam et al., 2000 ▶). Mukinda et al. (2007) ▶, concluded that aqueous extract of *Artemisia afra* was not toxic on the kidney tissue at a concentration of 1000, 2000 mg/kg in rat and mice, respective. Also, the amount of creatinine did not change in the treatment group. In another study which was done on female rat's reproductive system, exposure to Artemisia herba alba extract was not toxic at a concentration of 300 mg/kg for 4 weeks. Whereas, the extract of this plant was showed toxic effects at the same concentration for 12 weeks (Almasad et al., 2007 ▶). These results were not similar to the results of present study. Jayasimha Goud et al. (2011) ▶, also reported that *Artemisia absinthium *leaves methanol extract in different concentrations (100, 250 and 500 mg/kg) produced significant hypoglycemic activity, moreover, the extract of this plant reduced significantly the levels of urea and creatinine in diabetic rats. It can be probably due to hypoglycemic activity of this plant. Also, the effects of extract were increased in long-term treatment. In another study the extracts of Artemisia monosperma were studied against lipid peroxidation induced by lead acetate in rats. Lead administration increases significantly the amount of urea. But the extract of this plant reduced the elevated concentrations of urea to normal values. This protective effect may be due to high level of total antioxidant contents in this plant (Al-Soqeer, 2011 ▶). Ene-ojo et al. (2013) ▶, reported that two and six rats out of 24 died respectively at concentrations of 50, 100 mg/kg of chloroformic extract of *Artemisia maciverae*, whereas, at a concentration of 200 mg/kg all the animals died. Also, the kidney tissue was damaged in the treated groups with extract. Moreover, the levels of urea and creatinine were increased significantly in the 50, 100 and 200 mg/kg treatment groups compared to control group. These abnormalities were returned to normal when the treatment was finished. The observed changes may be attributed to the toxic effect of the plant extract that was dependent with dose and duration of treatment. So that, in the present study, the tissue destruction was more in the 200mg/kg group.

According to the findings of this study and other reports, it seems that the *A. deserti* flowering taps extract has toxic effects on kidney tissue, so that these effects were increased with increasing of concentration of extract. However, the reason for these toxic effects remains to be clarified and further studies are necessary.
